# Arctigenin Induces an Activation Response in Porcine Alveolar Macrophage Through TLR6-NOX2-MAPKs Signaling Pathway

**DOI:** 10.3389/fphar.2018.00475

**Published:** 2018-05-15

**Authors:** Zheng Lu, Lingling Chang, Qian Du, Yong Huang, Xiujuan Zhang, Xingchen Wu, Jie Zhang, Ruizhen Li, Zelin Zhang, Wenlong Zhang, Xiaomin Zhao, Dewen Tong

**Affiliations:** College of Veterinary Medicine, Northwest A&F University, Yangling, China

**Keywords:** arctigenin, porcine alveolar macrophage, NOX2, ROS, TLR6, MAPKs

## Abstract

Arctigenin (ARG), one of the most active ingredients abstracted from seeds of *Arctium lappa L.*, has been proved to exert promising biological activities such as immunomodulatory, anti-viral, and anti-cancer etc. However, the mechanism behind its immunomodulatory function still remains elusive to be further investigated. In this study, we found that ARG had no significant effects on the cell proliferation in both porcine alveolar macrophage cell line (3D4/21) and primary porcine derived alveolar macrophage. It remarkably increased the expression and secretion of the two cytokines including tumor necrosis factor-alpha (TNF-α) and transforming growth factor beta1 (TGF-β1) in a dose-dependent manner with the concomitant enhancement of phagocytosis, which are the indicators of macrophage activation. ARG also elevated the intracellular reactive oxygen species (ROS) production by activating NOX2-based NADPH oxidase. Furthermore, inhibition of ROS generation by diphenyliodonium and apocynin significantly suppressed ARG-induced cytokine secretion and phagocytosis increase, indicating the requirement of ROS for the porcine alveolar macrophage activation. In addition, TLR6-My88 excitation, p38 MAPK and ERK1/2 phosphorylation were all involved in the process. As blocking TLR6 receptor dramatically attenuated the NOX2 oxidase activation, cytokine secretion and phagocytosis increase. Inhibiting ROS generation almost abolished p38 and ERK1/2 phosphorylation, and the cytokine secretion could also be remarkably reduced by p38 and ERK1/2 inhibitors (SB203580 and UO126). Our finding gave a new insight of understanding that ARG could improve the immune-function of porcine alveolar macrophages through TLR6-NOX2 oxidase-MAPKs signaling pathway.

## Introduction

The immunomodulatory potentials of Chinese traditional medicine are gaining more interest to dip multiple research for prophylactic and therapeutic purposes in complex disorders caused by various etiological factors. The seeds derived from *Arctium lappa*, that containing arctigenin (ARG) as a main bioactive ingredient, have been used as a diuretic, anti-inflammatory and detoxifying agent in Chinese traditional medicine for centuries (Wu X. et al., 2014; Chang et al., 2015). Previous research have demonstrated that ARG has potent anti-tumor (Hsieh et al., 2014; Lu et al., 2015), anti-virus (Swarup et al., 2008; Chen et al., 2016), anti-oxidant (Predes et al., 2011; Wu R.-M. et al., 2014), cardiovascular and neuroprotective protection activities (Jang et al., 2002; Liu et al., 2015). Especially, it was found that ARG possesses immunomodulatory effects on macrophages. For example, ARG was proved to exhibit an anti-inflammatory activity via NF-κB or JAK-STAT pathway in LPS-stimulated RAW264.7 (Kang et al., 2008; Zhao et al., 2009; Kou et al., 2011). As reported by Hyam et al. (2013), ARG might ameliorate inflammatory diseases, such as colitis in rats, by inhibiting PI3K and polarizing M1 macrophages to M2-like macrophages. In another study, Xu et al. (2013) proved that ARG promoted cholesterol efflux in oxLDL-loaded THP-1 macrophages through upregulation of ABCA1, ABCG1 and apoE, which is dependent on the enhanced expression of PPAR-γ and LXR-α. Lately, ARG was found to alleviate the macrophages recruitment in the tubulointerstitium in a rat model of obstructive nephropathy, accompanied by the downregulation of NF-κB (p65) signal in the nuclear fraction and the decrease of pro-inflammatory mediator gene expression including MCP-1, TNF-α, IL-1β and IFN-γ in macrophages (Li et al., 2017). In most currently available research, ARG has been mainly demonstrated to resist inflammation *in vitro* and *in vivo*, however, the immune regulating effects and mechanism on macrophage in pigs have not been reported.

Macrophages are known as pleiotropic phagocytes that play an essential role in both innate and adaptive immunity. The main functions of macrophages include chemotaxis, phagocytosis, endocytosis, presenting antigens and secretion of cytokines that profoundly influence immune responses, all of which are integral to homeostasis, immune defense and tissue repair (Liu and Yang, 2013). Many types of research have suggested that macrophages could be activated and programmed into different phenotypes in response to various micro-environmental cues, including cytokines, antigen, drugs, and etc. Activated macrophages eliminate the pathogens, apoptotic cells, tumor cells and etc. by phagocytosis and gradually maturate of the phagosome into a phagolysosome where foreign intruders are attempted digested. Besides, they can also release a wide range of mediators, such as reactive oxygen species (ROS), nitric oxide (NO), hydrolytic enzymes, bioactive lipids, and pro-inflammatory cytokines (TNF-α, IL-1, IL-6, IL-12, and interferon etc.), indirectly or directly intensifying the lymphocytes function including natural killer (NK) cells, T cells and B cells (Zhang and Mosser, 2008). Currently, it is considered that successful immunotherapy will require immunity enhancement and/or immune-suppression decrease, thereby the modulation of macrophage activities is of great interest because it is a promising avenue explored in the therapeutic approaches to some diseases (Rodrigues and Grenha, 2015; Lundahl et al., 2017).

Increasing evidence indicates that macrophages are relatively quiescent until triggered by appropriate pathogen associated molecular patterns (PAMPs) that stimulate various pathogen recognition receptors (PRRs) expressed on macrophages (Zhang and Mosser, 2008). Toll-like receptors (TLRs) are one of the most thoroughly characterized PRRs, so far, over 10 members of the TLR family have been identified, which play a significant role in macrophage activation and participate in the first line of defense against invading pathogens (Ntoufa et al., 2016). TLR1/2/4/6 are located on the cytomembrane where they recognize mycobacterial lipoproteins, proteins and glycolipids (Lucas and Maes, 2013; Noreen and Arshad, 2015). TLR3/7/8/9 are expressed in endo-lysosomal compartments where they detect CpG DNA, single-stranded RNA and RNA (Wang et al., 2006; Tsai et al., 2009). Upon stimulation by respective PAMPs, TLR signaling pathway originates from the cytoplasmic Toll/IL-1 receptor (TIR) domain which recruit a set of TIR domain-containing adaptor protein (TIRAP) or TRIF-related adaptor molecule (TRAM) to signaling adapter molecules such as Myeloid differentiation primary response gene 88 (MyD88) and TIR-domain-containing adapter-inducing interferon-β (TRIF). Subsequently, MyD88 recruits IL-1R-associated kinases (IRAKs) to TLRs through the interaction of the death domains of both molecules. Phosphorylated IRAK-1 activates a multimeric protein complex composed of TRAF6, TAK1, TAB1 and TAB2, thereby leading to activation of MAPKs. Signaling cascades culminate in the nuclear translocation of transcription factors like nuclear factor (NF)-κB and interferon regulatory factors (IRFs) and subsequent production of inflammatory mediators, interferons and phagocytosis programs (Kawai and Akira, 2006; Moresco et al., 2011).

NADPH oxidases of the NOX family (NOX1-NOX5) are one of the important enzymatic sources of ROS, which catalyzes the NADPH dependent reduction of oxygen to superoxide (Kleniewska et al., 2012). Since activated macrophages produce a large volume of ROS through NADPH oxidase activation, which known as respiratory burst, ROS is considered as “activated macrophage marker” (Gwinn and Vallyathan, 2006). The NADPH oxidase consists of a membrane-bound flavocytochrome b558 (composed of p22phox and NOX) and four cytosolic subunits including p47phox, p67phox, p40phox, and small GTPase Rac (Kleniewska et al., 2012). Upon activation, the NADPH oxidase is initiated by two simultaneous mechanisms: the phosphorylation of p47phox on multiple sites and the activation of the small GTPase Rac2 followed by the migration of the cytosolic components to the membrane where they associate with the membrane-bound components to assemble the catalytically active oxidase (Brandes et al., 2014). It was reported that NOX families participate in ROS generation in response to diverse stimuli, resulting in the different cellular responses via activation of specific signaling pathways (Makni-Maalej et al., 2013; Zeng et al., 2014; Ding et al., 2016). And ROS production in macrophages has been found involved in activating signal transduction, such as MAPKs, NF-κB and AP-1, leading to expression of pro-inflammatory mediators (Kaul and Forman, 1996; Youn et al., 2016).

The present study was aimed to explore the effects of ARG on porcine alveolar macrophage immune functions including phagocytosis and cytokine secretion. Furthermore, our study elucidated the specific Toll-like receptor and NADPH oxidase, as well as detail signaling pathways involved in the activation of porcine alveolar macrophages in response to ARG. Our findings provide the evidence that ARG might have an immunomodulatory property to stimulate the innate and acquired immune responses in pigs.

## Materials and Methods

### Reagents

The RPMI 1640 medium, 100 × non-essential amino acids, 100 mM sodium pyruvate, penicillin, streptomycin and the ELISA kits were purchased from Invitrogen (Shanghai, China). Fetal bovine serum (FBS) were purchased from Gibco/BRL (Gaithersburg, MD, United States). Apocynin, diphenyleneiodonium (DPI), dimethyl sulfoxide (DMSO), SB203580 and UO126 were obtained from Sigma (St. Louis, MO, United States). Rabbit polyclonal primary antibodies of TLR6 was bought from Santa Cruz Biotechnology (Santa Cruz, CA, United States). Mouse monoclonal TLR6 blocking antibody (IgG) and TLR2/TLR6 agonist FSL-1 were bought from InvivoGen (San Diego, CA, United States). Rabbit polyclonal primary antibodies of p44/42 MAPK (Erk1/2), SAPK/JNK and MyD88, rabbit monoclonal primary antibody of p38 MAPK, phospho-p38 MAPK (Thr180/Tyr182) and phospho-p44/42 MAPK (Erk1/2) (Thr202/Tyr204), mouse polyclonal primary antibody of phospho-SAPK/JNK (Thr183/Tyr185) and anti-rabbit IgG HRP-linked antibody were bought from Cell Signaling Technology (Danvers, MA, United States). Rabbit polyclonal primary antibody of NOX2/gp91phox was bought from Absin Bioscience (Shanghai, China). Rabbit polyclonal primary antibody of p22phox was bought from Abcam (Cambridge, United Kingdom). Mouse polyclonal primary antibody of β-actin and anti-mouse IgG HRP-linked antibody were obtained from Invitrogen (Shanghai, China). All other chemicals and reagents used in this study were of highest quality and obtained from standard commercial sources.

Arctigenin (ARG) (purity ≥ 98%) purchased from JCKY Institute of Chemical Technology (Beijing, China) was dissolved at 20 mM with DMSO as a stock solution, stored at -20°C. Before all experiments, the desired concentrations of ARG were freshly diluted with medium from the stock.

### Cell Culture

Porcine alveolar macrophage cell line (3D4/21) was purchased from the American Type Culture Collection. The primary porcine alveolar macrophages were obtained from the lungs of SPF piglets as previously described (Chiou et al., 2000). Cells were maintained in RPMI 1640 medium supplemented with 10% fetal bovine serum, 100 IU/ml of penicillin, 100 μg/ml of streptomycin, 1× non-essential amino acids and 1 mM sodium pyruvate in a humidified incubator with 5% CO_2_ at 37°C.

### Cell Viability Assay

Exponentially growing 3D4/21 cells and primary porcine alveolar macrophages were seeded into 96-well plates at 2,000 cells per well. Following attachment the cells were exposed to various concentrations (0, 0.5, 1.0, 2.0 μM) of ARG in RMPI 1640 medium for the indicated time. Afterward, cell viability was analyzed using the Cell Counting Kit-8 (Beyotime, Jiangsu, China). Briefly, cells were stained with 100 μL fresh medium containing 10% CCK-8, and incubated at 37°C for 2 h. The absorbance was measured using a M200 PRO Micro-plate Reader (TECAN, Switzerland) at 450 nm. All experiments were performed in triplicates.

### Measurement of Intracellular Reactive Oxygen Species

The intracellular ROS was detected by using Reactive Oxygen Species Assay Kit (Beyotime Biotech., Jiangsu, China) according to the manufacturer’s instruction. After indicated treatment, 3D4/21 cells were washed twice and loaded with 10 μM DCFH-DA for 30 min at 37°C in the dark. The formation of the fluorescent-oxidized derivative of DCF-DA was monitored using a C6 Flow Cytometer (Becton Dickinson, United States) at the emission wavelength of 530 nm and an excitation wavelength of 485 nm. Finally, ROS generation was quantified by the median fluorescence intensity of 10,000 cells. All data were obtained in triplicate, independent experiments.

### Western Blotting Analysis

Cells were lysed in RIPA buffer. The protein concentration was determined using a BCA protein assay kit (Thermo scientific, United States) according to the manufacturer’s instructions. Whole cell lysates (40 μg protein) were resolved by 10% SDS-polyacrylamide gels electrophoresis and transferred onto PVDF membranes. Non-specific binding was blocked by Blocking Buffer (Beyotime Biotech., Jiangsu, China) for 3 h at 37°C. Subsequently, membranes were incubated with specific antibodies at an appropriate dilution overnight at 4°C. The membranes were washed with TBS with Tween-20 (TBS-T) and then incubated with HRP conjugated second antibodies. Specific complexes were visualized using Chemiluminescent HRP Substrate (Millipore Inc., Bedford, MA, United States). Densitometry was performed using the software Image J. All data were obtained in triplicate, independent experiments.

### Quantitative Real-Time PCR

Total RNA was extracted using Trizol (Invitrogen, Shanghai, China) according to the manufacturer’s protocol and reverse-transcribed into cDNA using PrimeScript^TM^ RT reagent Kit with gDNA Eraser (TaKaRa Bio Inc., Tokyo, Japan). Real-time PCR was performed in 96-well plates in a total reaction volume of 20 μl on Bio-Rad IQ5 Real-Time PCR System using SYBR^®^ Premix Ex Taq^TM^ II (TaKaRa Bio Inc., Tokyo, Japan). The relative expression level of mRNAs was normalized to that of the internal control β-actin using the 2^-ΔΔ*Ct*^ cycle threshold method. All experiments were independently performed three times and the average was used for comparison.

### Enzyme-Linked Immunosorbent Assay (ELISA)

The TNF-α protein level in the culture supernatants were determined using TNF alpha Porcine ELISA Kit (Thermo Scientific, United States), the IL-10 and TGF-β1 protein levels were determined using Porcine IL-10 Quantikine ELISA Kit and Mouse/Rat/Porcine/Canine TGF-beta 1 Quantikine ELISA Kit (R&D Systems, United States), respectively. Assays were performed according to the manufacturer’s instructions. All experiments were performed in triplicates, and the cytokines levels were calculated according to the standard curve.

### Small Interfering RNA (siRNA) Transfection

3D4/21 cells were plated on 6-well plates at 50% confluence before transfection. Individual siRNAs (at 10 nM), lipofectamine 2000 and Opti-MEM were mixed and incubated at room temperature for 20 min, siRNA-lipofectamine 2000 complexes were added to cells for 24 h and the medium was replaced by fresh medium after transfection. Knockdown effects by siRNA were confirmed by western blotting assay.

### Measurement of NADPH Oxidase Activity

After treatment, 3D4/21 macrophages were collected and centrifuged at 500 *g* for 5 min at 4°C, then re-suspended in PBS contented 250 μM NADPH and incubated at 37°C. NADPH consumption was assessed by monitoring the decrease in absorbance at wavelength 340 nm for 10 min. An aliquot of cells was lysed with 2% SDS and the protein content of cell lysates was estimated. Results were expressed as nmol of substrate/mg/minute of protein. All data were obtained in triplicate, independent experiments.

### Phagocytosis Assay

Phagocytosis assay was performed using pHrodo^TM^ Red Zymosan BioParticles^TM^ Conjugate (Life Technologies, Carlsbad, CA, United States). Briefly, after indicated treatments, the culture medium were replaced with 100 μL prepared pHrodo^TM^ BioParticles^TM^ (0.5 mg/mL). Then cells were transferred to an incubator and incubated at 37°C for 2 h. Fluorescence was measured at the emission wavelength of 585 nm and an excitation wavelength of 560 nm with Spectra Max M2 fluorescence microplate reader (Molecular Devices, United States). And the cells were visualized using Revolution WD confocal microscope (Andor, British). All experiments were performed in triplicates.

### Statistical Analysis

The statistical analysis was performed by one-way analysis of variance (ANOVA) using GraphPad prism 5.0 software by Dunnett’s test. Values are expressed as mean ± SD and all experiments were performed at least in triplicate. Cases in which *p* < 0.05 were considered statistically significant.

## Result

### Effect of ARG on the Proliferation of Porcine Alveolar Macrophage

**FIGURE 1:**
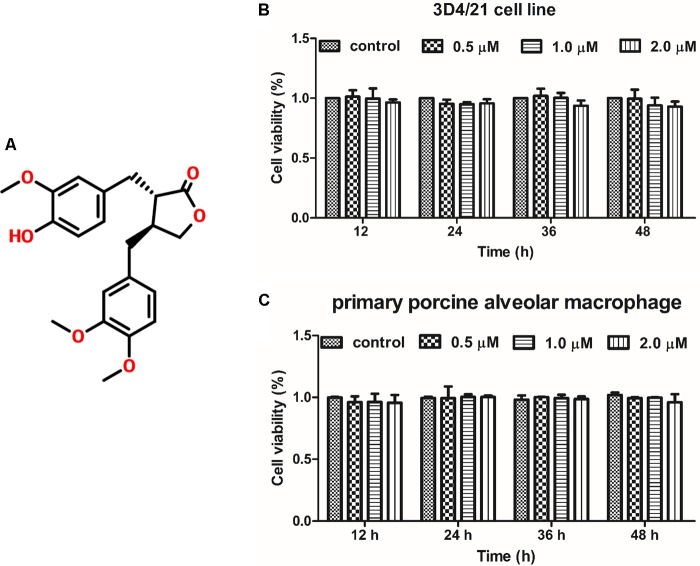
The effect of arctigenin (ARG) on the proliferation in porcine alveolar macrophages. **(A)** Chemical structure of ARG. **(B)** 3D4/21 cell line and **(C)** primary porcine alveolar macrophage were incubated with ARG (0, 0.5, 1.0, 2.0 μM) for 12, 24, 36, and 48 h. Cell viability was determined by Cell Counting Kit-8 (CCK-8) assay. Results are presented as means ± SD, *n* = 3. ^∗^*p* < 0.05, ^∗∗^*p* < 0.01, ^∗∗∗^*p* < 0.001 versus the control group.

To investigate the effect of ARG (the chemical structure of ARG was shown in **Figure 1A**) on cell proliferation in 3D4/21 cells and primary porcine alveolar macrophage, cells were incubated with ARG (0, 0.5, 1.0, and 2.0 μM) for 12, 24, 36, and 48 h, respectively. As shown in **Figures 1B,C**, ARG within 2 μM concentration showed neither proliferative nor anti-proliferative effects on both 3D4/21 cell line and primary porcine alveolar macrophages.

### ARG Induces an Activation Response in Porcine Alveolar Macrophage

**FIGURE 2:**
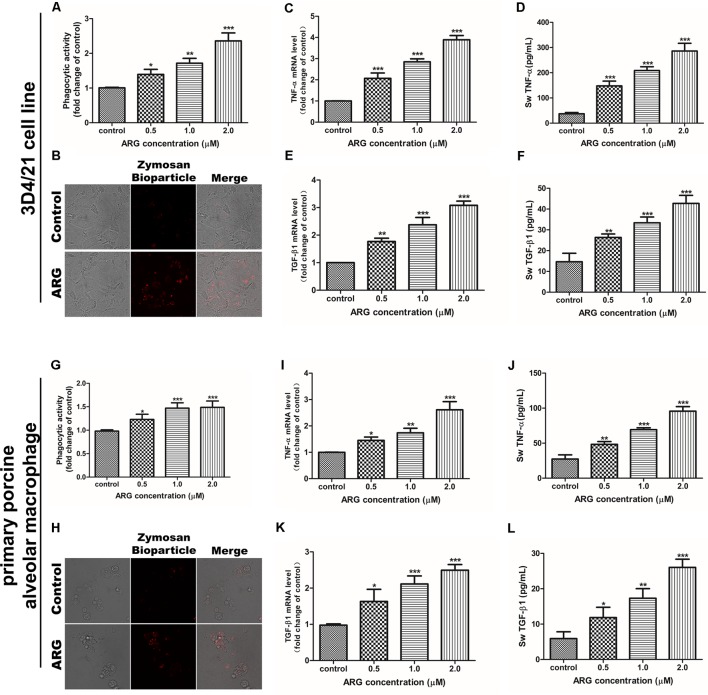
Effects of ARG on the phagocytosis, expressions and secretions of TNF-α and TGF-β1 in porcine alveolar macrophages. 3D4/21 cells and primary porcine alveolar macrophages were treated with ARG (0, 0.5, 1.0, 2.0 μM) for 24 h. **(A,G)** Phagocytic activity was determined using a phagocytosis assay. **(B,H)** Cells were visualized by confocal microscope (magnification 1000 × ). **(C,E,I,K)** Cells were lysed and total RNA was prepared to determine *TNF*-α and *TGF-β1* mRNA expression levels. **(D,F,J,L)** The amount of TNF-α and TGF-β1 secreted into the culture supernatant was determined by enzyme-linked immuno sorbent assay (ELISA). Results are presented as the means ± SD, *n* = 3. ^∗^*p* < 0.05, ^∗∗^*p* < 0.01, ^∗∗∗^*p* < 0.001 versus the control group.

We evaluated whether ARG-treatment is capable of increasing the phagocytic activity and the secretion of the three cytokines including TNF-α, IL-10 and TGF-β1 in porcine alveolar macrophage. According to **Figures 2A,G**, the dose-dependently improved phagocytic activity in both 3D4/21 cell line and primary porcine alveolar macrophage were observed with the increased concentrations of ARG, which could be further confirmed by fluorescence assay as shown in **Figures 2B,H**. On the other side, as shown in **Figures 2C,E,I,K**, ARG-treatment significantly increased the expression levels of *TNF*-α and *TGF-β1* mRNA in a dose-dependent manner. We further quantified the amount of TNF-α and TGF-β1 by ELISA assay in the culture supernatant (**Figures 2D,F,J,L**), both of which were significantly and concentration-dependently up-regulated during the ARG-treatment. Notably, ARG could not affect the expression or secretion of IL-10 (data not shown). These results suggested that ARG could induce a specific activation response in both 3D4/21 cell line and primary porcine alveolar macrophage, as the evidenced by the increased cytokine secretion and the phagocytic activity increase.

### ARG Increases the Generation of ROS by Activating NOX2-Based NADPH Oxidase

**FIGURE 3:**
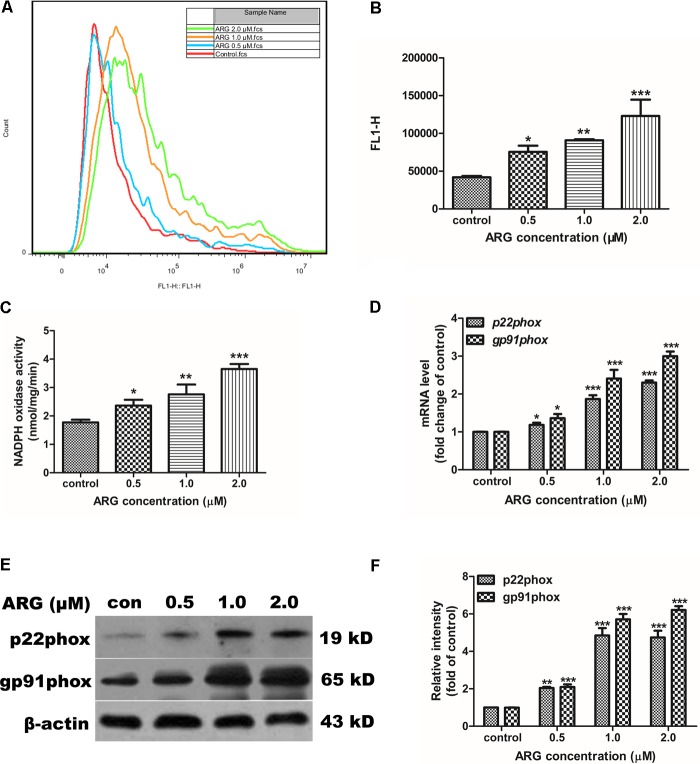
ARG elevated the ROS level in 3D4/21 macrophages by activating NOX2-based NADPH oxidase. 3D4/21 macrophages were treated with ARG (0, 0.5, 1.0, 2.0 μM) for 24 h. **(A,B)** The intracellular ROS level was analyzed by flow cytometry after loading with DCFH-DA, the representative histogram were shown. **(C)** Treated cells were harvested and incubated with 200 μM NADPH. NADPH consumption was monitored in absorbance at ° = 340 for 10 min, then cell extracts were analyzed for NADPH oxidase activity. **(D)** Total RNA was prepared for analyzing the mRNA expression level of NADPH oxidase subunits (*p22phox*, *gp91phox/NOX2*) by qRT-PCR using specific primers. **(E)** Cell extracts were analyzed for p22phox and gp91phox/NOX2 protein level by western blotting using specific antibodies, β-actin was employed as a loading control. **(F)** Relative protein levels of p22phox and gp91phox/NOX2 were quantified by scanning densitometry and normalized to β-actin levels. Data were presented as means ± SD, *n* = 3, and western blotting data are representative of three independent experiments. ^∗^*p* < 0.05, ^∗∗^*p* < 0.01, ^∗∗∗^*p* < 0.001 versus the control group.

To evaluate the effects of ARG on the intracellular ROS generation, 3D4/21 cells were incubated with ARG at the concentration of 0, 0.5, 1.0, and 2.0 μM for 24 h. According to **Figures 3A,B**, ARG-treatment remarkably increased the intracellular ROS generation in a dose-dependent manner. To gain insight into the possible mechanism how ARG mediated the ROS formation. NADPH oxidase activity was determined as described previously, which was also dramatically enhanced in a dose-dependent manner (**Figure 3C**). Furthermore, the expression levels of the NADPH oxidase subunits mRNA were shown in **Figure 3D**, ARG treatment resulted in a significant up-regulation of *NOX2/gp91phox* and *p22phox*, however, *p47phox*, *p67phox* and *p40phox* were unchanged (**Supplementary Figure S1**). Besides, in western blotting detection, ARG-treatment led to a remarkably dose-dependent increase of gp91phox and p22phox protein (**Figures 3E,F**). However, increased ROS level and NADPH oxidase activity that were almost eliminated when we knocked down the *gp91phox* and *p22phox*, respectively (**Supplementary Figures S3A,B**). Furthermore, the co-incubation with oxidase assembly inhibitor apocynin or flavoprotein inhibitor DPI could also significantly attenuate the ARG induced ROS generation and NADPH oxidase activity enhancement (**Supplementary Figures S3C,D**). These results indicated that ARG elevated the ROS level in 3D4/21 macrophage by activating NOX2-based NADPH oxidase.

### NOX2-Based NADPH Oxidase Depended ROS Generation Was Involved in 3D4/21 Macrophage Activation

**FIGURE 4:**
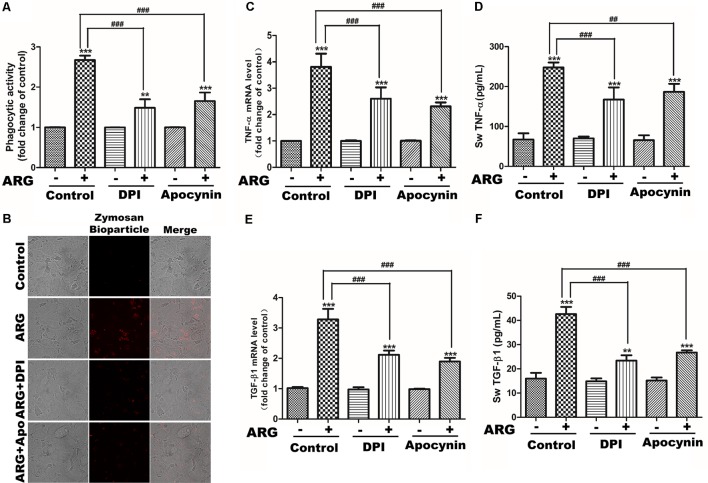
Effects of NADPH oxidase inhibitors on ARG-induced phagocytosis increase, expression and secretions of TNF-α and TGF-β1 in 3D4/21 macrophages. 3D4/21 macrophages were pretreated with 50 nM DPI or 100 μM apocynin for 2 h and treated with ARG (2.0 μM) for 24 h in the presence of DPI or apocynin. **(A)** Phagocytic activity was determined using a phagocytosis assay. **(B)** Cells were visualized by confocal microscope (magnification 1000×). **(C,E)** Cells were lysed and total RNA was prepared to determine *TNF*-α and *TGF-β1* mRNA expression level. **(D,F)** The amount of TNF-α and TGF-β1 secreted into the culture supernatant was determined by ELISA. Results are presented as the means ± SD, *n* = 3. ^∗^*p* < 0.05, ^∗∗^*p* < 0.01, ^∗∗∗^*p* < 0.001 versus control group, ^#^*p* < 0.05, ^##^*p* < 0.01, ^###^*p* < 0.001 versus ARG-treated only group.

We next investigated the role of NADPH oxidase depended ROS generation in cytokine secretion and phagocytosis. Apocynin and DPI were co-incubated with ARG, respectively. As shown in **Figures 4C,E**, the expression levels of *TNF-α and TGF-β1* mRNA were significantly suppressed compared to that ARG only treated group. Meanwhile, knocking down either *gp91phox* or *p22phox* (the knocking down effect was examined by western blotting in **Supplementary Figure S2A**) led to an expressional reduction of *TNF*-α and *TGF-β1* mRNA (**Supplementary Figures S2C,E**). Similarly, the secretion of TNF-α and TGF-β1were also remarkably decreased during the co-operation with NADPH oxidase inhibitors (apocynin and DPI) (**Figures 4D,F**) and the transfection with siRNAs (siRNA *p22phox* and siRNA *gp91phox*) (**Supplementary Figures S2D,F**). In addition, we observed that ARG-induced increase of phagocytic activity was efficiently suppressed by apocynin and DPI (**Figures 4A,B**), and was also attenuated via siRNA *p22phox* and siRNA *gp91phox* transfection (**Supplementary Figure S2B**). All the results indicated that NOX2 oxidase activation and ROS generation were required for ARG-induced activation in 3D4/21 macrophages.

### ARG Induced the Phosphorylation of p38 MAPK and ERK in 3D4/21 Macrophages

**FIGURE 5:**
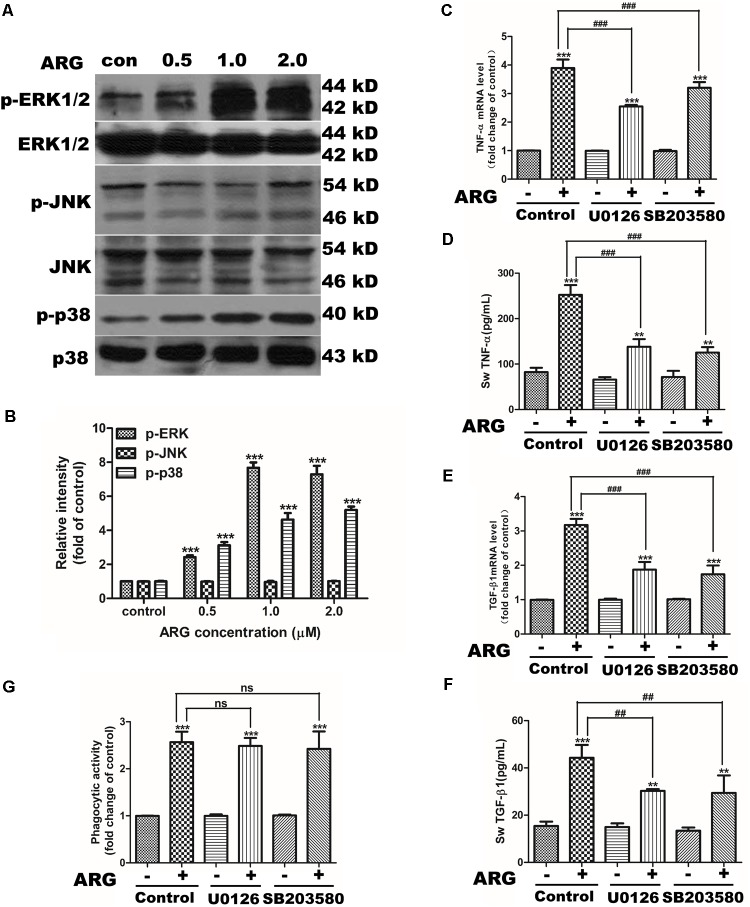
MAPK activation was involved in ARG-induced expression and secretions of TNF-α and TGF-β1 in 3D4/21 macrophages. 3D4/21 macrophages were treated with ARG (0, 0.5, 1.0, 2.0 μM) for 24 h. **(A)** Cell extracts were analyzed for p-p38 MAPK, p38 MAPK, p-JNK, JNK, p-ERK and ERK protein level by western blotting using specific antibodies. **(B)** Relative protein levels of p-p38 MAPK, p-JNK and p-ERK were quantified by scanning densitometry and normalized to total MAPK levels. 3D4/21 macrophages were pretreated with 20 μM SB203580 and 20 μM U0126 for 1 h and treated with ARG (2.0 μM) for 24 h in the presence of SB203580 and U0126. **(C,E)** Total RNA was prepared and analyzed for the expression level of *TNF*-α and *TGF-β1* mRNA by qRT-PCR. **(D,F)** TNF-α and TGF-β1 levels in the culture medium were measured by ELISA. **(G)** Phagocytic activity was determined using a phagocytosis assay. Data were presented as means ± SD, *n* = 3, and western blotting data are representative of three independent experiments. ^∗^*p* < 0.05, ^∗∗^*p* < 0.01, ^∗∗∗^*p* < 0.001 versus the control group, ^#^*p* < 0.05, ^##^*p* < 0.01, ^###^*p* < 0.001 versus ARG-treated only group.

To identify the effect of ARG on MAPKs activation, and whether MAPKs signaling pathway was involved in the ARG-induced activation of 3D4/21 macrophages. Cells were incubated with ARG alone or together with specific inhibitors. As shown in **Figures 5A,B**, ARG-treatment significantly increased the phosphorylation of ERK1/2 and p38 MAPK in a dose-dependent manner, however, JNK was unchanged. Suggesting that ARG is capable of activating MAPKs. In addition, UO126 and SB203580 were respectively used to inhibit the phosphorylation of ERK and p38 MAPK. And both the mRNA and protein level of TNF-α and TGF-β1 were found significantly decreased upon the inhibitors (**Figures 5C–F**). While the intensified phagocytic activity did not affect by the inhibition of ERK or p38 MAPK phosphorylation (**Figure 5G**). These results suggested that p38 and ERK phosphorylation were involved in ARG induced cytokine secretion but might not in charge of the phagocytic activity.

### NADPH Oxidase Depended ROS Generation Was the Key Step in MAPKs Activation

**FIGURE 6:**
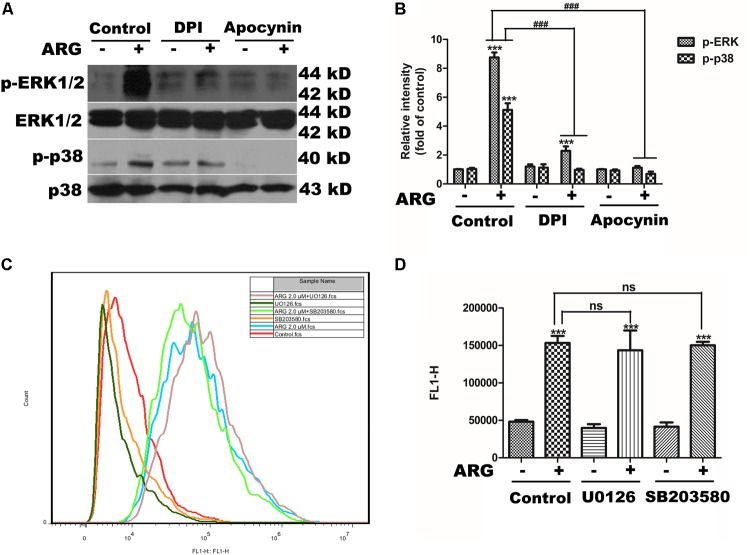
Reactive oxygen species (ROS) was the up-stream regulator of the MAPKs activation. 3D4/21 macrophages were pretreated with 50 nM DPI or 100 μM apocynin for 2 h and treated with ARG (2 μM) for 24 h in the presence of DPI or apocynin. **(A)** Cell extracts were analyzed for p-p38 MAPK, p38 MAPK, p-ERK and ERK protein level by western blotting using specific antibodies. **(B)** Relative protein levels of p-p38 MAPK and p-ERK were quantified by scanning densitometry and normalized to total MAPK levels. 3D4/21 macrophages were pretreated with 20 μM SB203580 and 20 μM U0126 for 1 h and treated with ARG (2 μM) for 24 h in the presence of SB203580 and U0126. **(C,D)** The intracellular ROS level was analyzed by flow cytometry after loading with DCFH-DA, the representative histogram were shown. Data were presented as means ± SD, *n* = 3, and western blotting data are representative of three independent experiments. ^∗^*p* < 0.05, ^∗∗^*p* < 0.01, ^∗∗∗^*p* < 0.001 versus the control group, ^#^*p* < 0.05, ^##^*p* < 0.01, ^###^*p* < 0.001 versus ARG-treated only group.

We observed that DPI or apocynin efficiently reduced the p38 MAPK and ERK activation in ARG-treated cells (**Figures 6A,B**). Furthermore, we also observed that UO126 and SB203580 did not affect ROS production (**Figures 6C,D**). Taken together, these results suggested that NADPH oxidase depended ROS generation was the key step in MAPKs activation induced by ARG in 3D4/21 macrophages.

### Critical Role of TLR6-MyD88 Pathway in the Activation of 3D4/21 Macrophage

**FIGURE 7:**
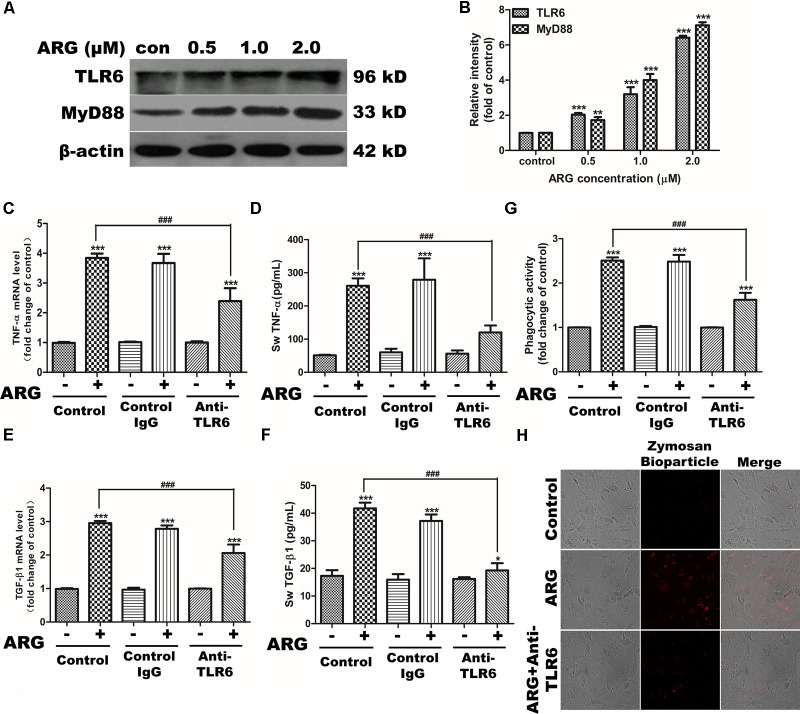
TLR6/MyD88 pathway activation were involved in ARG-induced phagocytosis increase, expression and secretions of TNF-α and TGF-β1 in 3D4/21 macrophages. 3D4/21 macrophages were treated with ARG (0, 0.5, 1.0, 2.0 μM) for 24 h. **(A)** Cell extracts were analyzed for TLR6 and MyD88 protein level by western blotting using specific antibodies, β-actin was employed as a loading control. **(B)** Relative protein levels of TLR6 and MyD88 were quantified by scanning densitometry and normalized to β-actin levels. Cells were pretreated with 2 μg/mL anti-TLR6 IgG monoclonal antibody for 1 h and treated with ARG (2.0 μM) for 24 h in the presence of anti-TLR6 IgG, using mouse IgG as control. **(C,E)** Total RNA was prepared and analyzed for the expression level of *TNF*-α and *TGF-β1* mRNA by qRT-PCR. **(D,F)** TNF-α and TGF-β1 level in the culture medium were measured by ELISA. **(G)** Phagocytic activity was determined using a phagocytosis assay. **(H)** Cells were visualized by confocal microscope (magnification 1000×). Data were presented as means ± SD, *n* = 3, and western blotting data are representative of three independent experiments. ^∗^*p* < 0.05, ^∗∗^*p* < 0.01, ^∗∗∗^*p* < 0.001 versus the control group, ^#^*p* < 0.05, ^##^*p* < 0.01, ^###^*p* < 0.001 versus ARG-treated only group.

To investigate whether TLRs were involved in the cell-activation mechanism of ARG, the mRNA expression of *TLRs* (*TLR1-10*) were detected in response to the ARG stimulation. As shown in **Supplementary Figure S4A**, the expression level of *TLR6* mRNA other than the other *TLRs* was significantly up-regulated. Then, we further investigated whether ARG induced macrophage-activation was associated with the TLR6-mediated immune responses. The data presented in **Figures 7A,B** showed that stimulation of 3D4/21 with ARG remarkably increased the protein expression of TLR6 and the adaptor molecule MyD88 in a dose-depend manner. However, knocking down either *TLR6* or *MyD88* using siRNA (the knocking effect was examined by western blotting in **Supplementary Figure S5A**) resulted in a considerable down-regulation of TNF-α and TGF-β1 in both mRNA and protein level (**Supplementary Figures S5C–F**), and also decreased the phagocytic activity in 3D4/21 macrophage (**Supplementary Figure S5B**). Our findings confirmed that the excitation of TLR6/MyD88 pathway played a critical role in the activation of 3D4/21 macrophage. To determine whether the target receptor of ARG was TLR6, we used neutralizing anti-TLR6 IgG antibody to block the TLR6 receptor (the blocking effect was detected by western blotting in **Supplementary Figure S4B**). Results of our study demonstrated that blocking TLR6 was able to diminish the cytokine expression and secretion (**Figures 7C–F**) and phagocytic activity increase (**Figures 7G,H**), suggesting that ARG might activate 3D4/21 macrophages by utilizing TLR6 as a receptor.

### TLR6 Activation Was Involved in Regulating NOX2-Based NADPH Oxidase Depended ROS Generation

**FIGURE 8:**
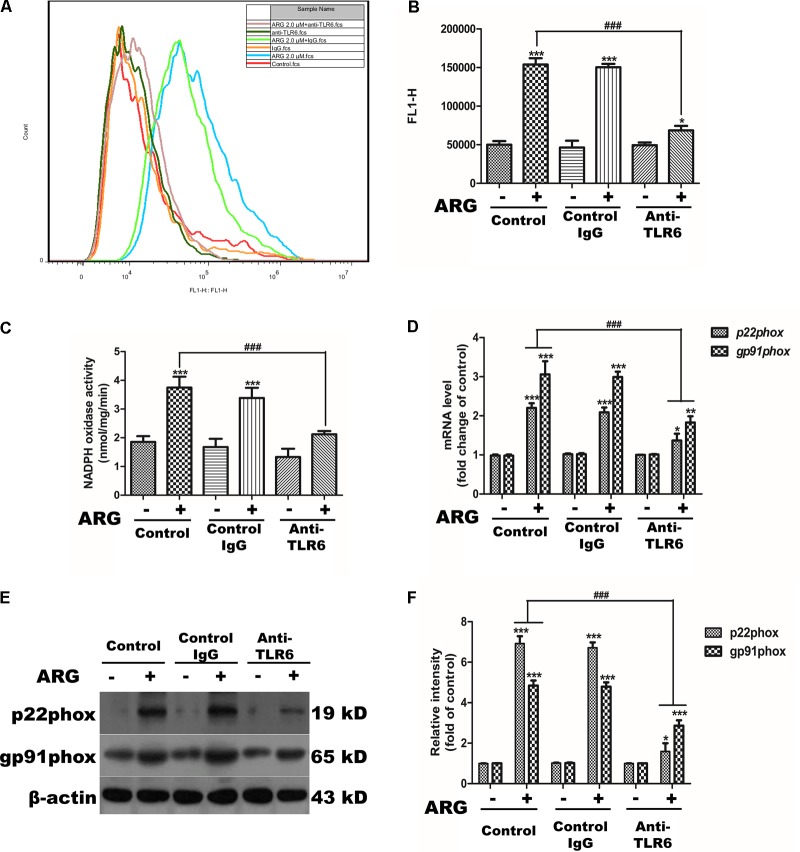
TLR6 activation was involved in regulating NOX2-based NADPH oxidase dependent ROS generation. 3D4/21 macrophages were pretreated with 2 μg/mL anti-TLR6 IgG monoclonal antibody for 1 h and treated with ARG (2.0 μM) for 24 h in the presence of anti-TLR6 IgG, using mouse IgG as control. **(A,B)** The intracellular ROS level was analyzed by flow cytometry after loading with DCFH-DA, the representative histogram were shown. **(C)** NADPH oxidase activity was analyzed as previously described. **(D)** Total RNA was prepared for analyzing the mRNA expression level of NADPH oxidase subunits (*p22phox*, *gp91phox/NOX2*) by qRT-PCR using specific primers. **(E)** Cell extracts were analyzed for p22phox and gp91phox/NOX2 protein level by western blotting using specific antibodies, β-actin was employed as a loading control. **(F)** Relative protein levels of p22phox and NOX2/gp91phox were quantified by scanning densitometry and normalized to β-actin levels. Data were presented as means ± SD, *n* = 3, and western blotting data are representative of three independent experiments. ^∗^*p* < 0.05, ^∗∗^*p* < 0.01, ^∗∗∗^*p* < 0.001 versus the control group, ^#^*p* < 0.05, ^##^*p* < 0.01, ^###^*p* < 0.001 versus ARG-treated only group.

We further investigated the sequence of events linking TLR6 to NOX2 oxidase activation. Monoclonal anti-TLR6 IgG antibody was used to prevent ARG from activating TLR6 receptor. As shown in **Figures 8A,B**, inhibiting the activation of TLR6 resulted in a remarkably decrease in the intracellular ROS generation. The NADPH oxidase activity was also determined dramatically attenuated compared to that ARG-treated only group (**Figure 8C**). The mRNA expression levels of the NADPH oxidase subunits (*NOX2/gp91phox* and *p22phox*) were shown in **Figure 8D**, anti-TLR6 IgG resulted in a significant down-regulation of *gp91phox* and *p22phox* mRNA compared to the ARG treated only group. Similar results were also found in protein levels of gp91phox and p22phox (**Figures 8E,F**). These above findings above revealed that TLR6 activation was involved in regulating NOX2 oxidase depended ROS generation.

## Discussion

A substantial amount of evidence have shown that the immune-regulatory activity of ARG had mainly been manifested in the anti-inflammatory effect targeting on murine macrophages (Zhao et al., 2009; Kou et al., 2011; Hyam et al., 2013; Li et al., 2017). As we found, different macrophages respond differently to ARG, which might depend on the species and genetic characteristics, or on the physiological/pathological state of macrophages. In current study, we firstly found ARG could induce an activation response in porcine alveolar macrophage without any cytotoxicity, which had never been reported before. However, the mouse derived macrophage was not activated the same as porcine derived macrophage in response to ARG under the same condition (data not shown). Hence, we supposed that ARG might have a functional promoting effect on porcine macrophages, and 3D4/21 cell line was chosen as a model to investigate the underlying mechanism.

It is well known that macrophages are central mediators in both innate and adaptive immunity, contributing to the initiation defensive reactions against pathogens and the resolution of inflammation. Previous published research proved that macrophages can be activated and programmed to several distinct subsets which have been broadly classified as M1 or M2 macrophages in response to various types of stimuli. M1 macrophages are potent effector cells eradicate invading microorganisms and tumor cells via phagocytosis and promote type I immune responses through the generation of a respiratory burst and production of pro-inflammatory cytokines. M2 macrophages are not only responsible for ameliorating inflammatory responses and adaptive immunity but also participate in regulating type 2 immune responses, wound healing and tissue repair (Liu and Yang, 2013). We found that ARG within 2 μM had no cytotoxic effect on 3D4/21 macrophages or primary porcine alveolar macrophage, but it could significantly increase the phagocytic activity which is one of the most distinguished features of activated macrophage. In addition, the expression and secretion of TNF-α and TGF-β1 were proved to be dose-dependently up-regulated, suggesting that the cytokine secretory capability of porcine alveolar macrophage was enhanced by ARG. All these findings indicating that ARG effectively induces an activation response in porcine alveolar macrophages.

According to our results, the intracellular ROS was elevated in a dose-dependent manner by ARG. To our best of understanding, there are multiple potential synthetic sources of ROS have already been found, such as NADPH oxidase, xanthine oxidase, lipoxygenase, mitochondrial electron transport and etc. (Paravicini and Touyz, 2008; Huang et al., 2016). Of these, NADPH oxidase activation and subsequent ROS production are considered as an important response to diverse stimuli during macrophage activation (Yu et al., 2014; Youn et al., 2016). We found DPI and apocynin, NADPH oxidase inhibitors, significantly inhibited ARG-induced ROS generation. Along with we also further demonstrated that ARG up-regulated expression of gp91phox and p22phox both in mRNA and protein level as well as NADPH oxidase enzymatic activity, suggesting NOX2-based NADPH oxidase is involved in ARG-induced ROS generation. However, DPI and apocynin are not specific for NOX2-based NADPH oxidase. Therefore, we knocked down gp91phox and p22phox using specific siRNAs. Results showed that siRNA *gp91phox* and siRNA *p22phox* almost abolished the ARG-induced ROS generation and NADPH oxidase enzymatic activity increase, indicating that the contribution of other NADPH oxidases and non-NADPH oxidases in ARG-induced ROS generation might be ruled out. Notably, ARG was also proved to increase the intracellular ROS level upon activating NOX2-based NADPH oxidase in human breast carcinoma cells according to the research from Hsieh et al. (2014). We supposed that ARG might have a specific stimulating effect on NOX2-based NADPH oxidase in some cell types. We further found that NOX2-based NADPH oxidase-dependent ROS generation is not only responsible for the secretion of cytokine but also for the phagocytic activity increase. It has been reported that ROS synthesized from NOX2-based NADPH oxidase contribute to killing microorganisms and tumor cells in activated macrophages (Elbim, 2005) and mediating a variety of biological functions as an intracellular messenger molecule (Yu et al., 2014; Youn et al., 2016). In this study, ROS form NOX2-based NADPH oxidase obviously act as an intracellular messenger molecule in the process of ARG-induced 3D4/21 macrophage activation. Similar results have been found in polyethylenimine-coated SPIONs triggered macrophage activation (Mulens-Arias et al., 2015) and *Ganoderma atrum* polysaccharide-induced TNF-α secretion during macrophage activation (Yu et al., 2014).

There are various kinds of immune-modulators including polysaccharide from plants (Sun et al., 2015; Xie et al., 2016), fungi (Liu et al., 2016; Wang et al., 2016) and algae (Cho et al., 2014; Ferreira et al., 2015), inactivated virus (Yang et al., 2017), and some bacteria etc. (Jung et al., 2015) that have been reported to be able to stimulate the immune system primarily by activating macrophages. Different immune-modulators exerted diverse response on macrophages, which depend on the binding receptors and the subsequent intracellular signal transduction. Furthermore studies revealed that evolutionarily conserved family of TLRs on play a critical role in identifying these immune-modulators and regulating the immunologic function of macrophages, as TLR4 and TLR2 have been proved to be the specific receptor for polysaccharide and lipoteichoic acid (a component of lactobacilli cell wall), respectively. In the present study, ARG selectively increased the expression of TLR6 and its down-stream adaptor molecule MyD88. Moreover, knocking down TLR6 and MyD88 led to an attenuation of both cytokine secretion and phagocytic activity increase, indicating that TLR6-MyD88 pathway was involved in the macrophage-stimulating mechanism. We further observed whether the cell-activating ability of ARG depended on TLR6 and whether ARG was directly recognized by TLR6. Our results showed that blocking TLR6 using anti-TLR6 antibody almost eliminated the macrophage-activating effect, indicating ARG is recognized by TLR6 and acts as a TLR6 agonist on 3D4/21 macrophages. In addition to ARG, (1,4)-α-D-glucan from *Tinospora cordifolia* activates the immune system through the activation of macrophages that occurs through TLR6 signaling, NF-κB translocation and cytokine production (Nair et al., 2006). Dengue virus NS1 protein has been proved to be responsible for the activation of TLR6 and TLR2 in DV-infected human PBMC (Chen et al., 2015). Unlike the macromolecular including polysaccharide, lipoteichoic acid, protein and etc., small lipo-soluble molecules like ARG are generally thought to be able to enter cells directly through cell membranes. But our data firstly proved that TLR6 might be the specific receptor for ARG. Besides, we also clarified that TLR6-MyD88 activation was responsible for NOX2-based NADPH oxidase activation in 3D4/21 macrophage. It is further confirmed that ARG activated 3D4/21 macrophages through binding to TLR6 instead of stimuli NADPH oxidase directly. And the respective role of ARG on TLRs in difference cell type needs to be further investigated.

It is well documented that various acts on TLR induce MyD88-mediated activation of MAPKs canonical pathway and consequently trigger the secretion of cytokines, and MAPK phosphorylation is a prerequisite for the cytokine production in activated macrophages (Yadav and Chandra, 2017). According to our data, p38 MAPK and ERK 1/2 were both found phosphorylated in response to ARG, and inhibiting the phosphorylation of either p38 MAPK or ERK 1/2 led to a remarkable decrease in the expression and secretion of TNF-α and TGF-β1. To further investigate the upstream pathway involved in the regulation of p38 MAPK and ERK 1/2, we focus on the effect of ROS on MAPKs pathway. Makni-Maalej et al. (2013) have demonstrated that MAPK phosphorylation took part in triggering the activation of NADPH oxidase. But some researchers prove that ROS can mediate the activation of MAPK pathways by a number of cellular stimuli in several cell types (Hsieh et al., 2014; Yu et al., 2014; Youn et al., 2016). Interestingly, Yang et al. (2011) challenge these concepts and reported that, the capsular polysaccharide of pyrogenic liver abscess *Klebsiella pneumonia* (PLAK. pneumoniae)-mediated activation of ERK1/2, JNK1/2, and p38 MAPK is independent of ROS. Our data showed that inhibition of ROS by DPI and apocynin diminished ARG-induced ERK1/2 and p38 MAPK phosphorylation, which suggested that ARG-mediated activation of ERK1/2 and p38 MAPK was dependent on ROS. However, the inhibitors of ERK1/2 and p38 MAPK had no effect on the NOX2-based NADPH oxidase activation or the ROS generation. All these results above demonstrated that NADPH oxidase activation was prior to the MAPKs phosphorylation and ROS was the up-stream regulating molecule. Unexpectedly, neither p38 MAPK nor ERK is responsible for the phagocytic activity increase, it is related to the TLR6-MyD88 activation.

## Conclusion

**FIGURE 9:**
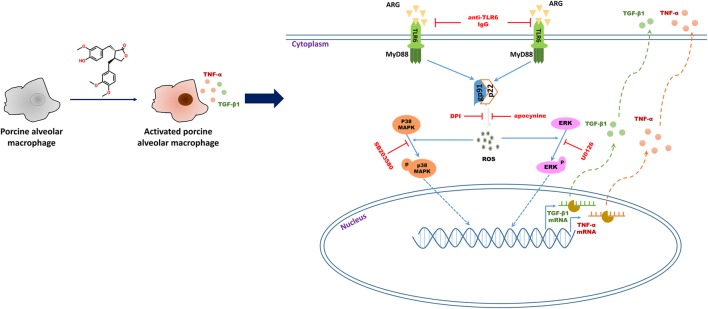
Schematic representation of the molecular mechanisms describing ARG-induced porcine alveolar macrophages activation response.

In summary, we demonstrated that ARG had potent immune-stimulatory activity *in vitro* and *in vivo*, as evidenced by cytokine secretion and phagocytosis activity increase in both 3D4/21 cell line and primary porcine alveolar macrophage. Further studies revealed that ARG induced 3D4/21 macrophage activation through NOX2 oxidase and MAPKs signaling pathways via the TLR6 receptor (as shown in **Figure 9**). Taken together, these results lead to a better understanding of the mechanisms that allow ARG to act as a potent adjuvant and immunomodulatory agent in pigs. We propose ARG as an activator to potentiate the host defense through TLR6 receptor.

## Ethics Statement

All animal experiments were approved by the Institutional Animal Care and Use Committee (IACUC) of Northwest A&F University (Permit Number: 20180405), and were performed according to the Animal Ethics Procedures and Guidelines of the People’s Republic of China. No other specific permissions were required for these activities. This study did not involve endangered or protected species.

## Author Contributions

ZL, QD, and LC developed the original idea, designed the experiments and elaborated the data. XjZ, XW, JZ, RL, and ZZ performed the experiments and prepared the figures. YH, XmZ, and WZ edited and reviewed the final version of the article. DT supervised the study. All listed authors contributed to article writing.

## Conflict of Interest Statement

The authors declare that the research was conducted in the absence of any commercial or financial relationships that could be construed as a potential conflict of interest. The reviewer Y-SB and handling Editor declared their shared affiliation.

## Supplementary Material

FIGURE S1 Effect of ARG on *p47phox*, *p67phox* and *p40phox* mRNA expression level. 3D4/21 macrophages were treated with ARG (0, 0.5, 1.0, 2.0 μM) for 24 h. Total RNA were prepared for analyzing the mRNA expression level of p47phox, p67phox and p40phox by qRT-PCR using specific primers. Data were presented as means ± SD, *n* = 3. ^∗^*p* < 0.05, ^∗∗^*p* < 0.01, ^∗∗∗^*p* < 0.001 versus the control group.

FIGURE S2 Effects of *p22phox* and *gp91phox* siRNA on ARG-induced phagocytosis increase, expression and secretion of TNF-α and TGF-β1 in 3D4/21 macrophages. 3D4/21 macrophages were transfected with specific *p22phox*, *gp91phox* siRNA and control siRNA, followed by treatment with ARG (2.0 μM) for 24 h. **(A)** The knocking-down effects of p22phox, gp91phox siRNA were detected by western blotting. **(B)** Phagocytic activity was determined using a phagocytosis assay. **(C,E)** Total RNA was prepared to determine *TNF*-α and *TGF-β1* mRNA expression levels. **(D,F)** The amount of TNF-α and TGF-β1 secreted into the culture supernatant were determined by ELISA. Results are presented as the means ± SD, *n* = 3. ^∗^*p* < 0.05, ^∗∗^*p* < 0.01, ^∗∗∗^*p* < 0.001 versus control group, ^#^*p* < 0.05, ^##^*p* < 0.01, ^###^*p* < 0.001 versus ARG-treated only group.

FIGURE S3 The specific stimulating effect of ARG on NOX2-based NADPH oxidase. 3D4/21 macrophages were transfected with specific *p22phox*, *gp91phox* siRNA and control siRNA, followed by treatment with ARG (2.0 μM) for 24 h. **(A)** The intracellular ROS level was analyzed by flow cytometry after loading with DCFH-DA. **(B)** NADPH oxidase activity was analyzed as described. Cells were pretreated with DPI or apocynin for 2 h and treated with ARG (2.0 μM) for 24 h in the presence of DPI or apocynin. **(C)** The intracellular ROS level was analyzed by flow cytometry after loading with DCFH-DA. **(D)** NADPH oxidase activity was analyzed as described. Results are presented as the means ± SD, *n* = 3. ^∗^*p* < 0.05, ^∗∗^*p* < 0.01, ^∗∗∗^*p* < 0.001 versus control group, ^#^*p* < 0.05, ^##^*p* < 0.01, ^###^*p* < 0.001 versus ARG-treated only group.

FIGURE S4 Effect of ARG on *TLR1-10* mRNA expression level. **(A)** 3D4/21 macrophages were treated with 2.0 μM ARG for 24 h. Total RNA was prepared for analyzing the mRNA expression level of *TLR1-10* by qRT-PCR using specific primers. Data were presented as means ± SD, *n* = 3. ^∗^*p* < 0.05, ^∗∗^*p* < 0.01, ^∗∗∗^*p* < 0.001 versus the control group. **(B)** 3D4/21 macrophages were treated with FSL-1 (TLR6 agonist) with or without neutralizing anti-TLR6 IgG antibody for 24 h. MyD88 expression level was detected by western blotting, β-actin was employed as a loading control.

FIGURE S5 Effects of *TLR6* and *MyD88* siRNA on ARG-induced phagocytosis increase, expression and secretions of TNF-α and TGF-β1 in 3D4/21 macrophages. 3D4/21 macrophages were transfected with specific *TLR6*, *MyD88* siRNA and control siRNA, followed by treatment with ARG (2.0 μM) for 24 h. **(A)** The knocking-down effects of *TLR6*, *MyD88* siRNA were detected by western blotting. **(B)** Phagocytic activity was determined using a phagocytosis assay. **(C,E)** Total RNA was prepared to determine *TNF*-α and *TGF-β1* mRNA expression levels. **(D,F)** The amount of TNF-α and TGF-β1 secreted into the culture supernatant were determined by ELISA. Results are presented as the means ± SD, *n* = 3. ^∗^*p* < 0.05, ^∗∗^*p* < 0.01, ^∗∗∗^*p* < 0.001 versus control group, ^#^*p* < 0.05, ^##^*p* < 0.01, ^###^*p* < 0.001 versus ARG-treated only group.

## Abbreviations

ARG: arctigenin
DAF-FM DA: 4-amino-5-methylamino-α2′,7′-difluorescein diacetate
DCFH-DA: ?2′,7′-dichlorodihydrofluorescein diacetate
DMSO: dimethyl sulfoxide
DPI: diphenylene iodide
ELISA: enzyme-linked immunosorbent assay
ERK: extracellular activated signal-regulated kinase
FBS: fetal bovine serum
JNK: c-Jun N-terminal kinase
MAPK: mitogen-activated protein kinase
MyD88: myeloid differentiation primary response gene 88
NADPH oxidase: nicotinamide adenine dinucleotide phosphate oxidase
ROS: reactive oxygen species
TGF-ββ1: transforming growth factor beta1
TLR6: toll-like receptor 6
TNF-α: tumor necrosis factor-alpha
