# Supplementary figures and images for: Arctigenin Induces an Activation Response in Porcine Alveolar Macrophage Through TLR6-NOX2-MAPKs Signaling Pathway
Source: Front Pharmacol. 2018 May 15;9:475. doi: 10.3389/fphar.2018.00475 (PMC5962800; doi:10.3389/fphar.2018.00475)

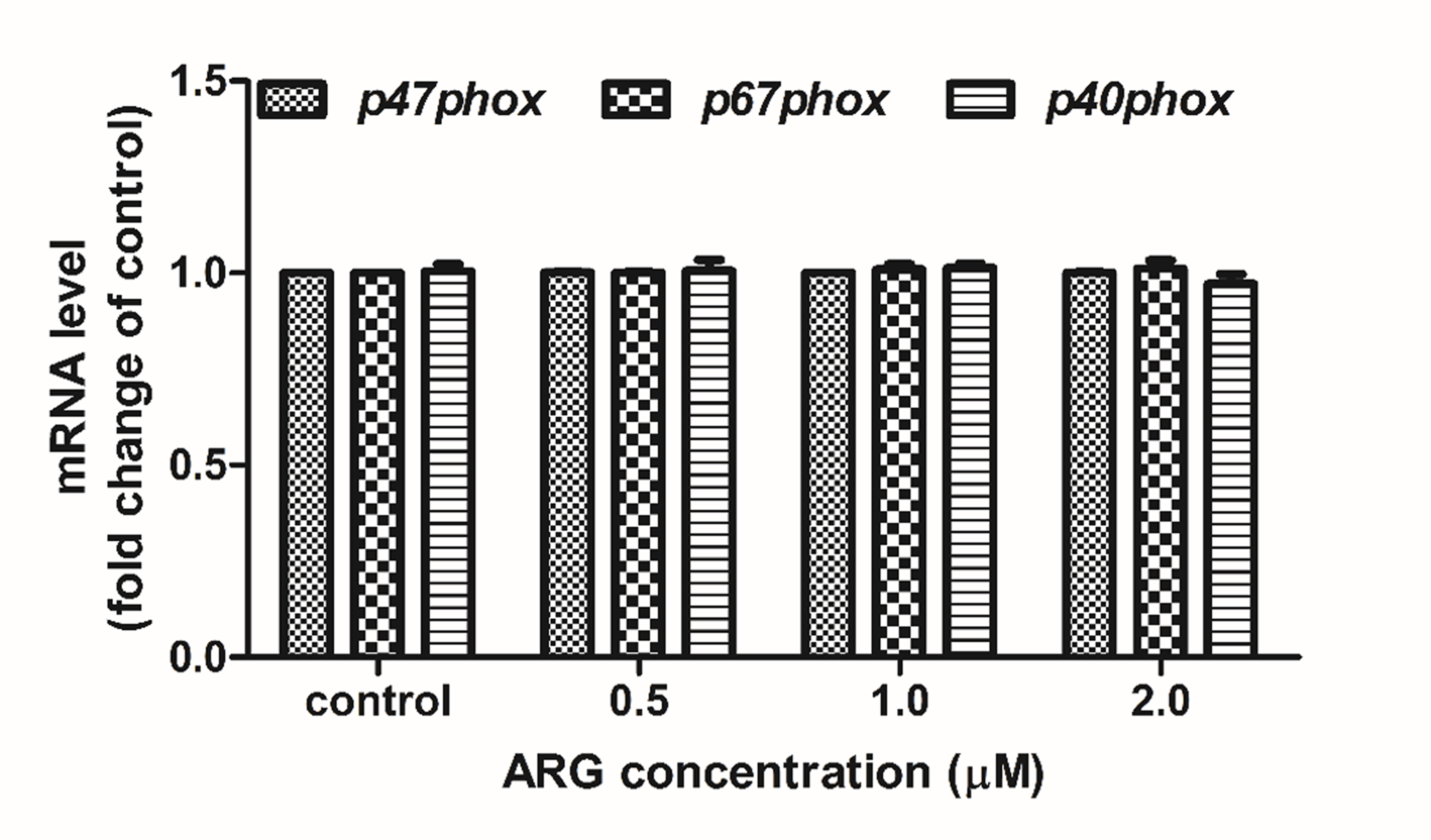

Supplement: FIGURE S1 — Effect of ARG on p47phox, p67phox and p40phox mRNA expression level. 3D4/21 macrophages were treated with ARG (0, 0.5, 1.0, 2.0 μM) for 24 h. Total RNA were prepared for analyzing the mRNA expression level of p47phox, p67phox and p40phox by qRT-PCR using specific primers. Data were presented as means ± SD, n = 3. ∗p < 0.05, ∗∗p < 0.01, ∗∗∗p < 0.001 versus the control group. [file Image_1.TIF]

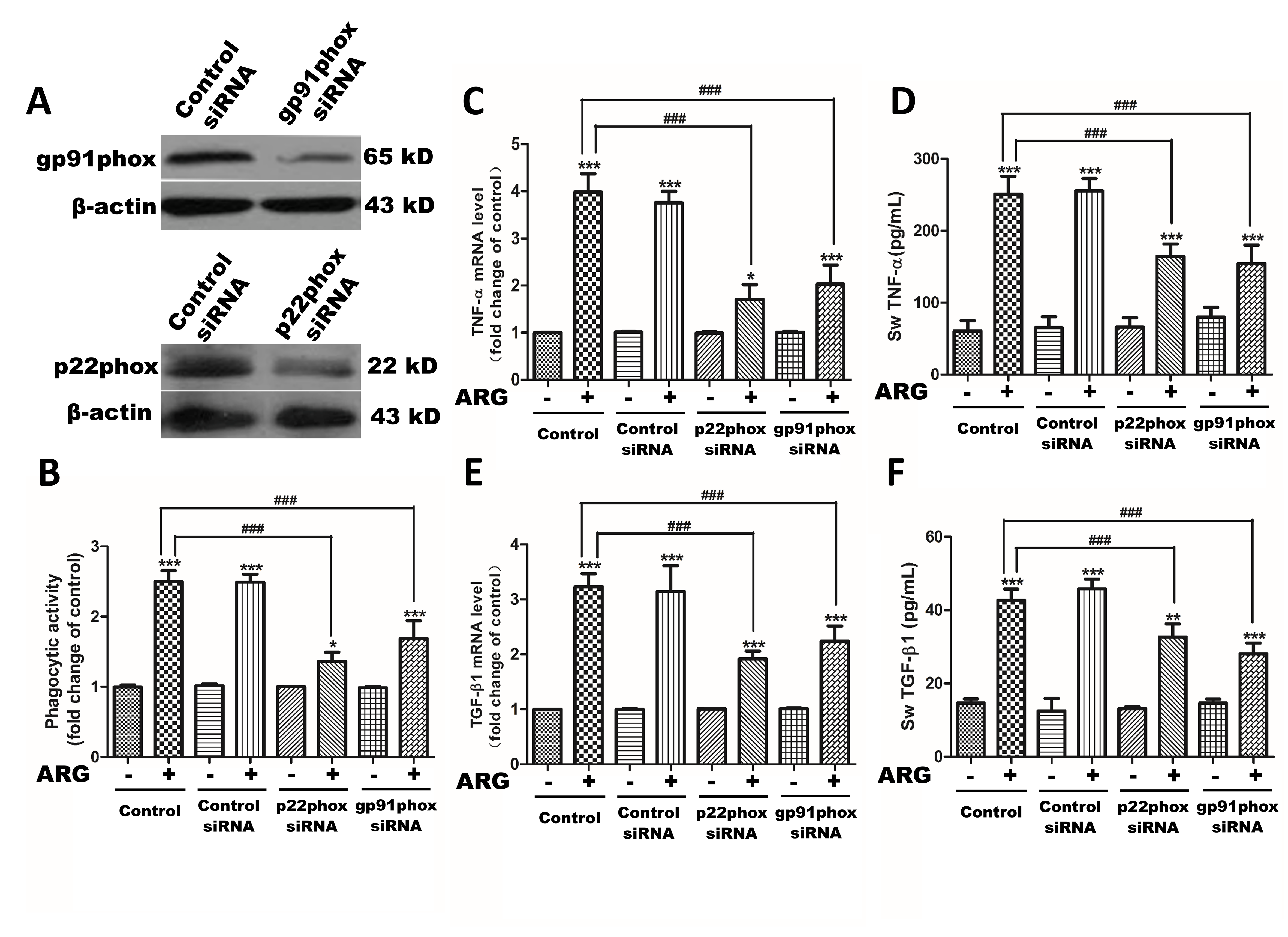

Supplement: FIGURE S2 — Effects of p22phox and gp91phox siRNA on ARG-induced phagocytosis increase, expression and secretion of TNF-α and TGF-β1 in 3D4/21 macrophages. 3D4/21 macrophages were transfected with specific p22phox, gp91phox siRNA and control siRNA, followed by treatment with ARG (2.0 μM) for 24 h. (A) The knocking-down effects of p22phox, gp91phox siRNA were detected by western blotting. (B) Phagocytic activity was determined using a phagocytosis assay. (C,E) Total RNA was prepared to determine TNF-α and TGF-β1 mRNA expression levels. (D,F) The amount of TNF-α and TGF-β1 secreted into the culture supernatant were determined by ELISA. Results are presented as the means ± SD, n = 3. ∗p < 0.05, ∗∗p < 0.01, ∗∗∗p < 0.001 versus control group, #p < 0.05, ##p < 0.01, ###p < 0.001 versus ARG-treated only group. [file Image_2.TIF]

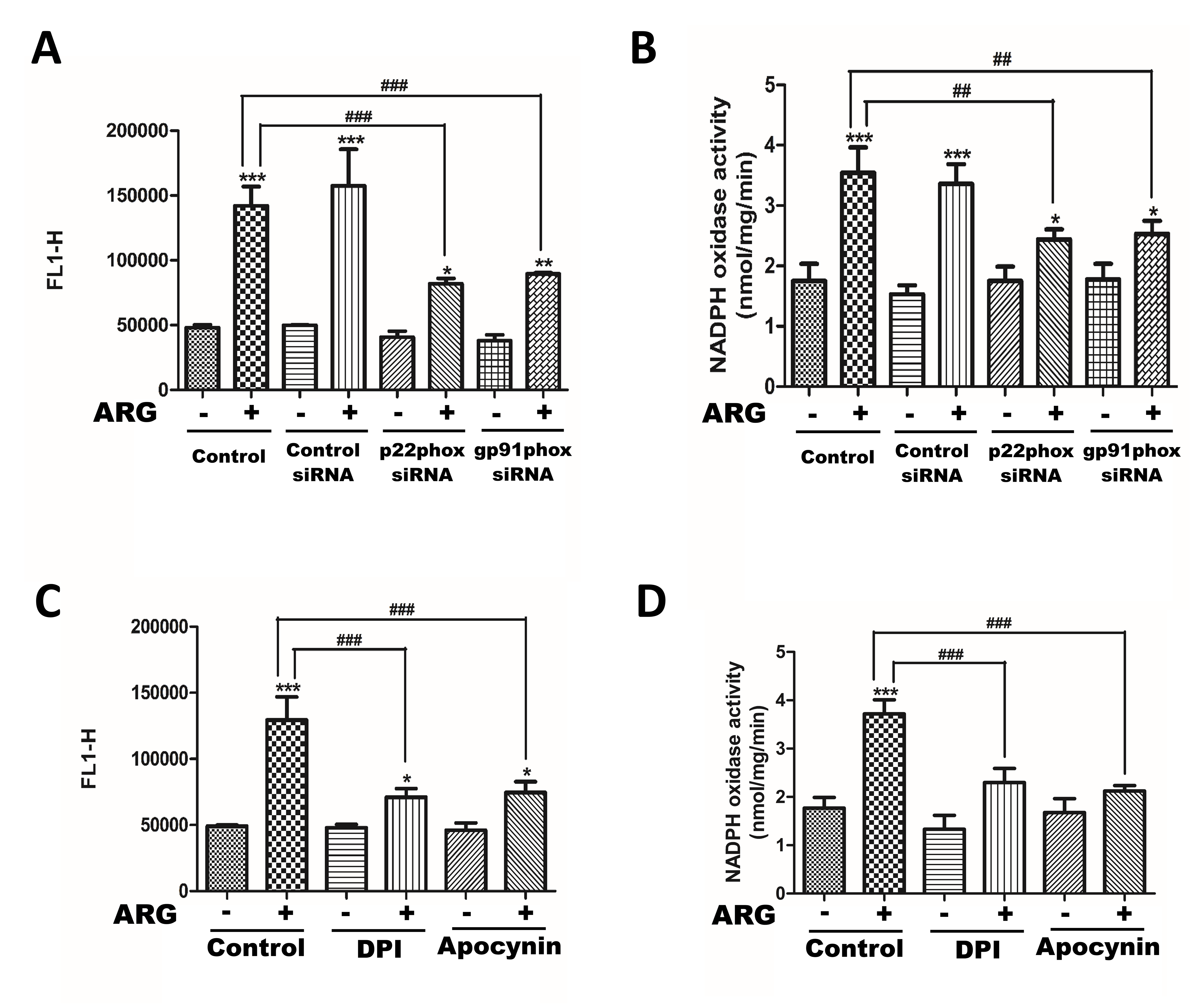

Supplement: FIGURE S3 — The specific stimulating effect of ARG on NOX2-based NADPH oxidase. 3D4/21 macrophages were transfected with specific p22phox, gp91phox siRNA and control siRNA, followed by treatment with ARG (2.0 μM) for 24 h. (A) The intracellular ROS level was analyzed by flow cytometry after loading with DCFH-DA. (B) NADPH oxidase activity was analyzed as described. Cells were pretreated with DPI or apocynin for 2 h and treated with ARG (2.0 μM) for 24 h in the presence of DPI or apocynin. (C) The intracellular ROS level was analyzed by flow cytometry after loading with DCFH-DA. (D) NADPH oxidase activity was analyzed as described. Results are presented as the means ± SD, n = 3. ∗p < 0.05, ∗∗p < 0.01, ∗∗∗p < 0.001 versus control group, #p < 0.05, ##p < 0.01, ###p < 0.001 versus ARG-treated only group. [file Image_3.TIF]

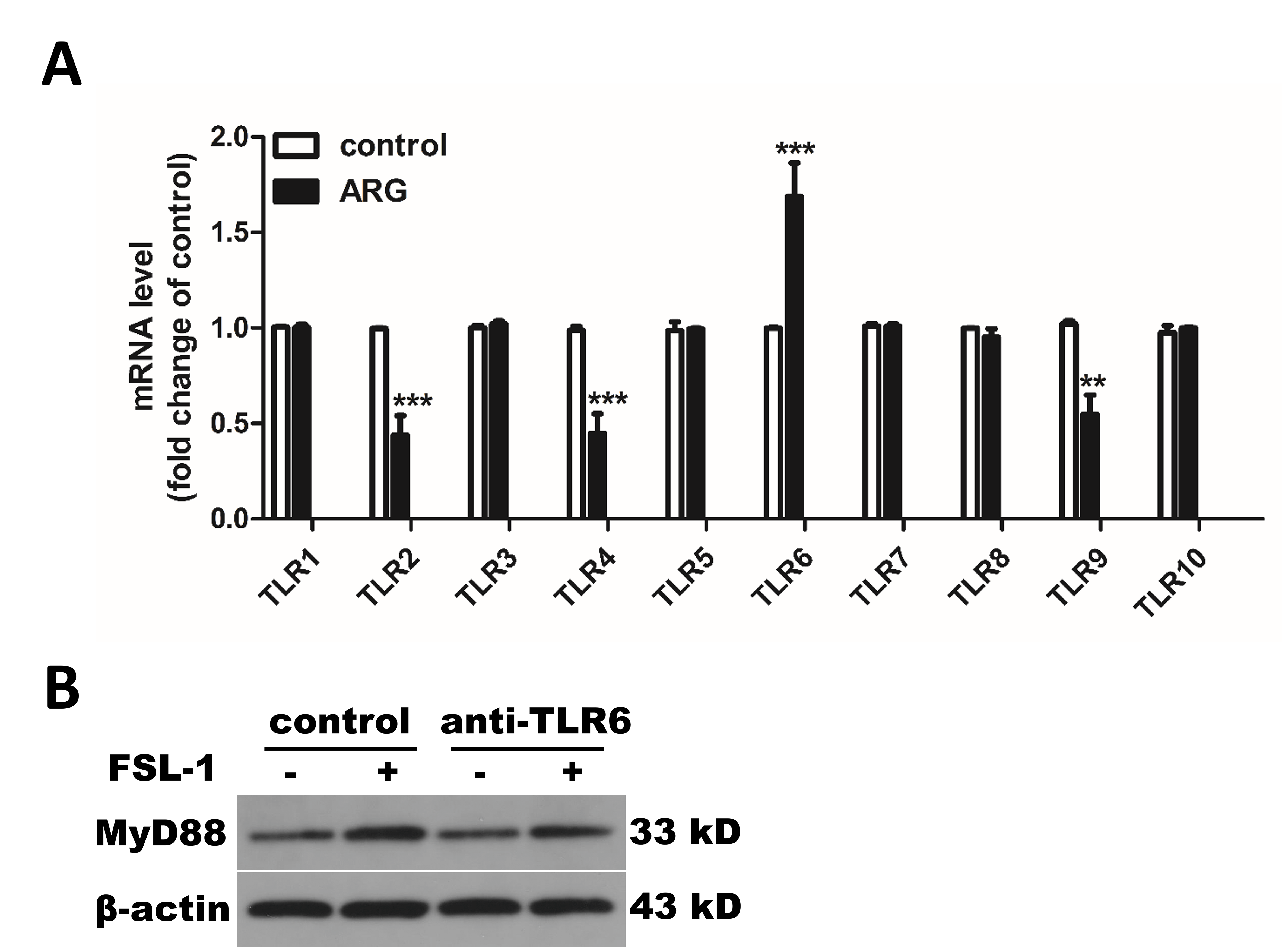

Supplement: FIGURE S4 — Effect of ARG on TLR1-10 mRNA expression level. (A) 3D4/21 macrophages were treated with 2.0 μM ARG for 24 h. Total RNA was prepared for analyzing the mRNA expression level of TLR1-10 by qRT-PCR using specific primers. Data were presented as means ± SD, n = 3. ∗p < 0.05, ∗∗p < 0.01, ∗∗∗p < 0.001 versus the control group. (B) 3D4/21 macrophages were treated with FSL-1 (TLR6 agonist) with or without neutralizing anti-TLR6 IgG antibody for 24 h. MyD88 expression level was detected by western blotting, β-actin was employed as a loading control. [file Image_4.TIF]

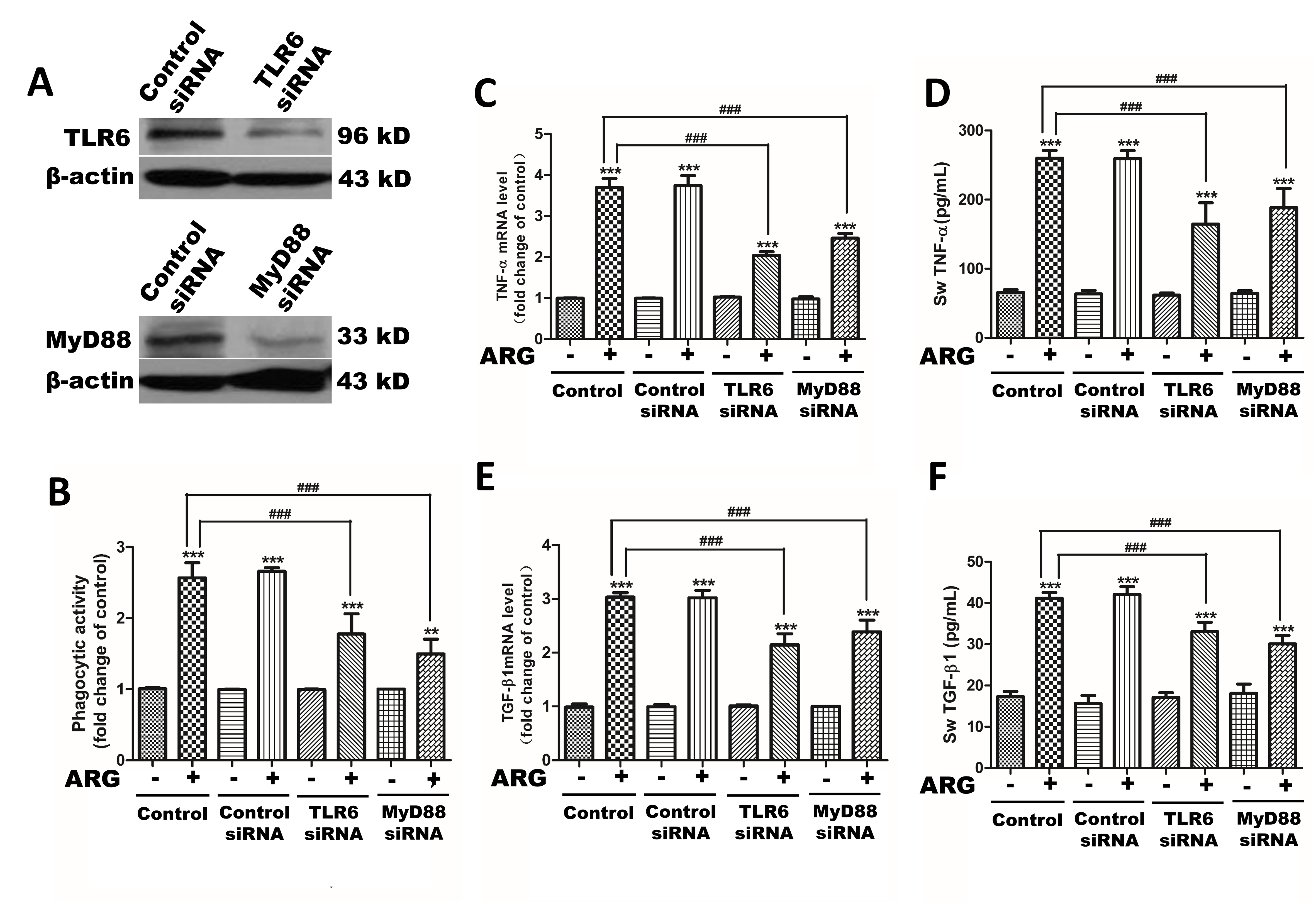

Supplement: FIGURE S5 — Effects of TLR6 and MyD88 siRNA on ARG-induced phagocytosis increase, expression and secretions of TNF-α and TGF-β1 in 3D4/21 macrophages. 3D4/21 macrophages were transfected with specific TLR6, MyD88 siRNA and control siRNA, followed by treatment with ARG (2.0 μM) for 24 h. (A) The knocking-down effects of TLR6, MyD88 siRNA were detected by western blotting. (B) Phagocytic activity was determined using a phagocytosis assay. (C,E) Total RNA was prepared to determine TNF-α and TGF-β1 mRNA expression levels. (D,F) The amount of TNF-α and TGF-β1 secreted into the culture supernatant were determined by ELISA. Results are presented as the means ± SD, n = 3. ∗p < 0.05, ∗∗p < 0.01, ∗∗∗p < 0.001 versus control group, #p < 0.05, ##p < 0.01, ###p < 0.001 versus ARG-treated only group. [file Image_5.TIF]
